# Proposed virulence-associated genes of *Streptococcus suis* isolates from the United States serve as predictors of pathogenicity

**DOI:** 10.1186/s40813-021-00201-6

**Published:** 2021-02-28

**Authors:** April A. Estrada, Marcelo Gottschalk, Aaron Rendahl, Stephanie Rossow, Lacey Marshall-Lund, Douglas G. Marthaler, Connie J. Gebhart

**Affiliations:** 1 Department of Veterinary and Biomedical Sciences, College of Veterinary Medicine, University of Minnesota, Saint Paul, MN USA; 2 Faculty of Veterinary Medicine, University of Montreal, Saint-Hyacinthe, Quebec Canada; 3 Veterinary Diagnostic Laboratory, College of Veterinary Medicine, University of Minnesota, Saint Paul, MN USA; 4 Veterinary Diagnostic Laboratory, College of Veterinary Medicine, Kansas State University, Manhattan, KS USA

**Keywords:** *Streptococcus suis*, Virulence-associated genes (VAGs), Pathotype, Pan-genome, Genetic diversity

## Abstract

**Background:**

There is limited information on the distribution of virulence-associated genes (VAGs) in U.S. *Streptococcus suis* isolates, resulting in little understanding of the pathogenic potential of these isolates. This lack also reduces our understanding of the epidemiology associated with *S. suis* in the United States and thus affects the efficiency of control and prevention strategies*.* In this study we applied whole genome sequencing (WGS)-based approaches for the characterization of *S. suis* and identification of VAGs.

**Results:**

Of 208 *S. suis* isolates classified as pathogenic, possibly opportunistic, and commensal pathotypes, the genotype based on the classical VAGs (*epf*, *mrp*, and *sly* encoding the extracellular protein factor, muramidase-release protein, and suilysin, respectively) was identified in 9% (*epf*+/*mrp*+/*sly*+) of the pathogenic pathotype. Using the chi-square test and LASSO regression model, the VAGs *ofs* (encoding the serum opacity factor) and *srtF* (encoding sortase F) were selected out of 71 published VAGs as having a significant association with pathotype, and both genes were found in 95% of the pathogenic pathotype. The *ofs*+/*srtF*+ genotype was also present in 74% of ‘pathogenic’ isolates from a separate validation set of isolates.

Pan-genome clustering resulted in the differentiation of a group of isolates from five swine production companies into clusters corresponding to clonal complex (CC) and virulence-associated (VA) genotypes. The same CC-VA genotype patterns were identified in multiple production companies, suggesting a lack of association between production company, CC, or VA genotype.

**Conclusions:**

The proposed *ofs* and *srtF* genes were stronger predictors for differentiating pathogenic and commensal *S. suis* isolates compared to the classical VAGs in two sets of U.S. isolates. Pan-genome analysis in combination with metadata (serotype, ST/CC, VA genotype) was illustrated to be a valuable subtyping tool to describe the genetic diversity of *S. suis.*

**Supplementary Information:**

The online version contains supplementary material available at 10.1186/s40813-021-00201-6.

## Background

The severe clinical presentation associated with *Streptococcus suis* infection is of increasing concern in the U.S. swine industry. The heterogeneity of *S. suis* can be described by serotyping and multi-locus sequence typing (MLST), and currently 29 true serotypes (1–19, 21, 23–25, 27–31, and 1/2) and 1551 registered sequence type (ST) profiles (as of December 2020) exist [1–5]. The numerous *S. suis* serotypes and STs limit our attempts to understand the epidemiology of the disease in an effort to prevent and manage the various clinical manifestations. Further*, S. suis* has zoonotic potential, and many of the effective antibiotics available for treatment are of high or critically important status per U.S. Food and Drug Administration’s Guidance for Industry #152 [6]. Also, serotype variations make it difficult to compare isolates within and across geographically distinct pig populations. The development of effective universal vaccines is hindered by the number of different virulent serotypes and the lack of knowledge of serotype- or ST-specific virulence markers and associated clinical disease.

Historically, systematic characterization of *S. suis* isolates occurred more extensively in other countries compared to the United States. For instance, serotypes 2, 3, and 1/2 have been well-characterized in Canadian swine populations [7–9]. In addition, virulence-associated genes (VAGs) (*epf*, *mrp*, and *sly*) and STs indicative of virulence potential were identified, mostly for serotype 2 [9–12]. Experimental studies illustrating the virulence potential of Canadian serotype 2 strains (ST1, ST25, and ST28) suggest the virulence potential of ST28 strains is low at best in Canada, but a very different clinical presentation was being observed on U.S. swine farms with ST28 [11, 13, 14]. In the past 4 to 5 years, *S. suis* infections on U.S. swine farms appeared to be more persistent and severe [15, 16]. However, whether this is a result of new circulating strains, an increase in virulence, or some other cause is not well understood, reinforcing the importance of the characterization of *S. suis* to monitor changes in strains within a herd.

U.S. swine practitioners utilize herd vaccination strategies as a means of controlling *S. suis* disease. However, selecting representative isolates and properly timing the administration of vaccines still remain a challenge [17]. As a result of the diversity of *S. suis*, limited commercial vaccines are available, and many practitioners develop and maintain farm-specific autogenous vaccines. In addition, clear criteria for identifying pathogenic strains that cause primary disease are lacking, making isolate selection for vaccines more difficult [18]. Isolates are commonly selected based on criteria such as serotype and isolation from systemic tissues [19, 20]. However, due to the diversity within and between serotypes, cross-protection between, and even within, different serotypes is difficult to attain [21–24]. Moreover, the presence of virulence markers is critical for selecting isolates for autogenous vaccines. Over 100 putative and confirmed virulence factors and markers (not crucial or critical for virulence) for *S. suis* have been described in the literature, but few have been verified in experimental models [18, 25]. These include Eurasian serotype 2 virulence markers extracellular protein factor (*epf* gene), muramidase-released protein (*mrp* gene), and suilysin (*sly* gene), which have been investigated in STs 1, 25, and 28 strains from North America [11, 26, 27].

The application of genomic approaches to identify associations between VAGs and disease manifestation can lead to a better understanding of *S. suis* pathogenesis. However, a comparative genomic study investigating the current distribution of *S. suis* VAGs in U.S. isolates is lacking. Recently, we reported associations of various pathotypes with subtypes, including serotype and ST, of *S. suis* [28]*.* In this current study, a genomic approach was utilized to identify associations between VAGs and pathotype of U.S. isolates while evaluating the likelihood of classical Eurasian serotype 2 VAGs and newly proposed VAGs to identify pathogenic strains. In addition, pan-genome genetic relationships, along with their VAG profiles (virulence-associated genotypes), were investigated for isolates within and between swine production companies. Finally, we applied the genomic approach for identifying associations between VAGs and pathogenicity classification to a validation set of *S. suis* isolates to determine whether our approach was robust enough to identify pathogenic strains isolated from other swine production companies.

## Materials and methods

### Source of isolates and collection of epidemiological data

A training set of 208 *S. suis* isolates were used in this study. These isolates, previously described by Estrada et al. (2019), were classified into three pathotypes (pathogenic, possibly opportunistic, and commensal) based on clinical information and site of isolation. “Pathogenic” isolates were obtained from systemic tissues such as the brain/meninges and heart. “Possibly opportunistic” isolates were predominantly from lung samples from pigs without signs of neurological or systemic disease. “Commensal” isolates were from laryngeal, tonsil, or nasal samples retrieved from farms with no current control methods for *S. suis* disease.

Furthermore, epidemiological data, such as swine production company and site, were collected for the training set of isolates. The swine production companies coded as A, D, E, K, and M are all large operations that range in size from 70,000 to 340,000 sows and with headquarters in the United States (A and D = MN, E = MO, K = KS, and M = IL).

### VAG profiling

VAG profiling was performed on the training set using a custom database of previously published VAGs of *S. suis* (Additional file 1) [28]. Illumina sequencing reads were mapped to reference DNA sequences (≥ 60% coverage and ≥ 90% sequence identity) using the SRST2 (Short Read Sequence Typing for Bacterial Pathogens) program [29]. The construction of a presence and absence heatmap (Euclidian distances and UPGMA clustering) was performed with R software [30].

### Statistical analysis

Associations between published *S. suis* VAGs and pathotype, as previously defined by Estrada et al. [28], were investigated. Published VAGs present in a majority of isolates (> 90%, 188/208) were removed. VAGs present in < 50% of the pathogenic pathotype (< 70/139) were also removed. Remaining VAGs were tested by chi-square, comparing the three pathotypes and the status (presence/absence) of individual genes. Genes lacking a significant (chi-square *p*-value < 0.05) association with pathotype were removed from the analysis. The remaining genes were analyzed using the Least Absolute Shrinkage and Selection Operator (LASSO) regression model [31].

The LASSO regression model reduces coefficients to zero and gradually eliminates genes that have no or low correlation with the target variable. The LASSO model was used to determine VAGs that may serve as the ‘best’ predictors of pathogenicity, in this case using the pathogenic pathotype as the indicator of pathogenicity. The analysis was performed using the R package glmnet and the best lambda penalty value to determine the fewest number of predictor genes [32]. Due to variation in the number of predictor VAGs in each run, we ran 100 iterations of the LASSO model to determine the most relevant predictor genes. Predictor VAGs reported in all 100 iterations were considered relevant candidate VAGs.

### Genome assembly and pan-genome analysis

Genome assembly was performed on Illumina sequencing data from the training set [28]: SRA accession numbers SRR9123061-SRR9123268. Genome assemblies were generated using MEGAHIT *de-novo* assembler (k-mer range of 25–225) and polished using Pilon [33, 34]. QUAST was used to evaluate the genome assemblies [35]. Only contigs that were 500 bp or larger were kept for annotation by Prokka to predict coding sequences [36]. The pan-genome was annotated using Roary with a 90% BLASTp identity cut-off to define clusters of genes and allowing paralog clustering [37, 38]. The FastTree program was used to generate an approximately-maximum-likelihood phylogenetic tree based on the binary presence and absence of core and accessory genes. Percent similarity was calculated as the percentage of shared genes in the pan-genome.

### Selection and whole genome sequencing of validation set

Thirty-two isolates obtained from a single swine production company from 2017 to 2019 were classified as either ‘pathogenic’ or of ‘unknown-pathogenicity’ based on tissue source (Additional file 2). Isolates classified as ‘pathogenic’ were obtained from the brain (*n* = 19). The isolates of ‘unknown-pathogenicity’ were isolated from non-systemic tissues (no neurological signs) (*n* = 13). The *S. suis* isolates were sequenced and the sequencing reads were processed using a similar method as described for the training set [28]. Isolates were confirmed as *S. suis* if they possessed the *S. suis*-specific recombination/repair protein (*recN*) sequence (*Streptococcus suis* 05HAS68, Accession CP002007).

### Serotype, MLST, VAG profile, and pan-genome analysis of validation set

The serotyping of the validation set of *S. suis* isolates was verified using a *S. suis* serotyping pipeline described by Athey et al. (2016) to differentiate serotypes 2 and 1/2 and serotypes 1 and 14 [39]. *In-silico* MLST analysis was performed using the SRST2 program, and the ST allele sequences and profiles obtained from the *S. suis* MLST database [5]. Novel STs were further grouped into major clonal complexes (CCs) as previously described [28]. Data on presence or absence of the classical VAGs (*epf*, *mrp,* and *sly*) was obtained for each of the 32 isolates as described above for the training set. Similar genome assembly and pan-genome analysis as described for the training set were performed on the 32 isolates. The number of gene clusters identified for the training and validation sets may differ due to gene duplication, pseudogenes, gene acquisition/loss, and other genomic variations, as well as differences in the number of genomes included in the pan-genome analysis [40, 41].

## Results

### VAG profiling

#### Distribution of the *epf*, *mrp*, and *sly* genes

In our previous study of 208 *S. suis* isolates (referred to as the training set), 139, 47, and 22 were classified as the pathogenic, possibly opportunistic, and commensal pathotype, respectively [28]. The training set was also characterized by determination of serotype, MLST, and CC. In the current study, the distribution of the *epf*, *mrp*, and *sly* genes was bioinformatically determined for the 208 isolates in the training set. These classical VAGs *epf*, *mrp*, and *sly* were identified in 20 (14.4%), 127 (91.4%), and 77 (55.4%) isolates of the pathogenic pathotype, respectively (Table 1). The *epf* gene was predominantly present in serotypes 1, 2, and 14 and CC1 isolates while *mrp* and *sly* were distributed among a diverse set of subtypes. The *epf*, *mrp*, and *sly* genes were identified in 0 (0%), 6 (27.3%), and 4 (18.2%) isolates of the commensal pathotype, respectively (Table 2). We further investigated genotype combinations of the *epf*, *mrp*, and *sly* genes and their distributions in STs 1, 25, and 28 (Table 2). The predominant genotype in the pathogenic pathotype was *epf*−/*mrp*+/*sly*- (41.0%, 57/139) followed by *epf*−/*mrp*+/*sly*+ (36.0%, 50/139). A majority of the ST28 (94%) and both ST25 isolates in the training set possessed the *epf*−/*mrp*+/*sly*- genotype. The *epf*+/*mrp*+/*sly*+ genotype was identified in only 20 of the 139 isolates classified as the pathogenic pathotype. All 17 ST1 isolates possessed the *epf*+/*mrp*+/*sly*+ genotype. In summary, a majority of isolates, even those of the pathogenic pathotype, lacked the three classical VAGs, but all the isolates containing the three VAGs were classified as pathogenic or ST1.

**Table 1:** Classical VAGs identified in the pathogenic pathotype of *S. suis* isolates (*n* = 139)

Subtypes			Percentage of positive		
			epf (***n*** = 20, 14.4%)	mrp (***n*** = 127, 91.4%)	sly (***n*** = 77, 55.4%)
**Serotypes**		1 (*n* = 11)	35.0	5.5	14.3
		1/2 (*n* = 54)	5.0	35.4	3.9
		2 (*n* = 17)	30.0	11.0	7.8
		3 (*n* = 18)	0.0	7.9	10.4
		4 (*n* = 8)	0.0	3.1	5.2
		5 (*n* = 13)	0.0	6.3	10.4
		6 (*n* = 2)	0.0	0.0	0.0
		7 (*n* = 23)	5.0	13.4	20.8
		8 (*n* = 8)	0.0	3.1	5.2
		9 (*n* = 8)	0.0	1.6	2.6
		10 (*n* = 3)	0.0	0.0	0.0
		14 (*n* = 5)	25.0	3.9	6.5
		23 (*n* = 10)	0.0	6.3	10.4
		24 (*n* = 1)	0.0	0.8	1.3
		1or14^a^ (*n* = 1)	0.0	0.0	1.3
		NT (*n* = 11)	0.0	1.6	0.0
**Multilocus Sequence Type**	**CC1**^**b**^	ST1 (*n* = 17)	85.0	13.4	22.1
	**CC1**	ST87 (*n* = 9)	0.0	3.1	5.2
	**CC28**	ST25 (*n* = 2)	0.0	1.6	0.0
	**CC28**	ST28 (*n* = 52)	10.0	33.1	3.9
	**CC28**	ST29 (*n* = 2)	0.0	1.6	0.0
	**CC28**	ST117 (*n* = 2)	0.0	1.6	0.0
	**CC28**	ST961 (*n* = 10)	0.0	7.1	1.3
	**CC28**	ST973 (*n* = 1)	0.0	0.8	0.0
	**CC94**	ST94 (*n* = 18)	0.0	11.0	18.2
	**CC94**	ST108 (*n* = 17)	0.0	11.0	18.2
	**CC94**	ST119 (*n* = 2)	0.0	0.8	1.3
	**CC94**	ST373 (*n* = 5)	0.0	3.1	5.2
	**CC94**	ST839 (*n* = 1)	0.0	0.8	1.3
	**CC94**	ST964 (*n* = 1)	0.0	0.8	1.3
	**CC94**	ST977 (*n* = 9)	0.0	5.5	9.1
	**CC94**	ST981 (*n* = 1)	0.0	0.8	1.3
	**CC104**	ST225 (*n* = 3)	5.0	0.8	3.9
		ST13 (*n* = 5)	0.0	0.0	6.5
		ST949 (*n* = 1)	0.0	0.0	0.0
		ST965 (*n* = 1)	0.0	0.0	0.0
		ST967 (*n* = 1)	0.0	0.0	0.0
		ST976 (*n* = 1)	0.0	0.0	0.0
		ST979 (*n* = 1)	0.0	0.8	1.3
		ST995 (*n* = 1)	0.0	0.0	0.0
		NF (*n* = 4)	0.0	2.4	0.0

^a^ Could not differentiate serotypes 1 and 14 by coagglutination, PCR, and WGS

^b^ Clonal complexes (CCs) determined in our previous study [27]

NT = Unresolved serotype by coagglutination, PCR, and WGS

NF = ST could not be determined

**Table 2:** Classical VAGs *epf*, *mrp*, and *sly* genotypes identified in the pathotypes of *S. suis* isolates (*n* = 208)

Virulence-associated gene or genotype	No. possessing gene or genotype (%)	Pathogenic (***n*** = 139)	Possibly Opportunistic (***n*** = 47)	Commensal (***n*** = 22)	ST1 (***n*** = 17)	ST25 (***n*** = 2)	ST28 (***n*** = 52)
epf	21 (10.1%)	20	1	0			
mrp	165 (79.3%)	127	32	6			
sly	99 (47.6%)	77	18	4			
epf−/mrp+/sly-	73 (35.1%)	57	14	2	0	2	49
epf−/mrp+/sly+	71 (34.1%)	50	17	4	0	0	1
epf−/mrp−/sly-	36 (17.3%)	5	15	16	0	0	0
epf+/mrp+/sly+	21 (10.1%)	20	1	0	17	0	2
epf−/mrp−/sly+	7 (3.4%)	7	0	0	0	0	0
epf+/mrp+/sly-	0	0	0	0	0	0	0
epf+/mrp−/sly+	0	0	0	0	0	0	0
epf+/mrp−/sly-	0	0	0	0	0	0	0

#### Determining predictors of pathotype by VAG profiling

Given the limited distribution of classical VAGs (*epf* and *sly*) among isolates in the pathogenic pathotype, the classical VAGs are not appropriate indicators of pathogenicity for non-serotype 2 *S. suis* isolates from the United States. Thus, a total of 71 previously published *S. suis* VAGs (including *epf*, *mrp*, and *sly*) were investigated for the presence of alternative genes that may be indicators of pathogenic strains. Thirty-two (45%) VAGs were present in all genomes regardless of pathotype and were clearly not indicators of the pathogenic pathotype (Table 3 & Additional file 3). Five VAGs were absent in all of the isolates in the commensal pathotype. *SalK* and *salR*, which encode the SalK/SalR two-component signal transduction system [42], were not detected in any of the isolates despite mapping to different reference sequences.

**Table 3:** Distribution of 71 VAGs for the pathotypes of *S. suis* isolates (*n* = 208)

VAG	No. containing the VAG	Pathogenic (***n*** = 139)	Possibly Opportunistic (***n*** = 47)	Commensal (***n*** = 22)
adcR^a^	208	139	47	22
amylopullulanase^a^	208	139	47	22
ccpA^a^	208	139	47	22
cdd^a^	208	139	47	22
ciaH^a^	208	139	47	22
dpr^a^	208	139	47	22
eno^a^	208	139	47	22
fbpS^a^	208	139	47	22
feoB^a^	208	139	47	22
gdh^a^	208	139	47	22
glnH^a^	208	139	47	22
gnd^a^	208	139	47	22
gpmA^a^	208	139	47	22
guaA^a^	208	139	47	22
guaB^a^	208	139	47	22
htpS^a^	208	139	47	22
lgt^a^	208	139	47	22
lpp^a^	208	139	47	22
orf207^a^	208	139	47	22
pepXP^a^	208	139	47	22
permease^a^	208	139	47	22
pgdA^a^	208	139	47	22
plr-gapA^a^	208	139	47	22
purA^a^	208	139	47	22
purD^a^	208	139	47	22
scrB^a^	208	139	47	22
scrR^a^	208	139	47	22
sodA^a^	208	139	47	22
srtA^a^	208	139	47	22
sspA^a^	208	139	47	22
troA^a^	208	139	47	22
vicR-covR^a^	208	139	47	22
arcA	207	139	46	22
ciaR	207	139	46	22
lmb	207	139	46	22
luxS	207	139	46	22
phospholipaseC	207	139	46	22
treR	207	139	46	22
ssnA	206	139	45	22
lspA	196	139	44	13
manN	196	139	43	14
zmpC	193	139	42	12
fur	190	136	41	13
gtfA	204	135	47	22
dltA^b^	186	135	39	12
ofs^b^	176	135	34	7
sao	189	134	39	16
GBSSAG0907-homologue^b^	175	133	35	7
srtF^b^	168	132	31	5
srtF-sipF^b^	167	131	31	5
mrp^b^	165	127	32	6
srtF-sfp1^b^	155	120	31	4
hylA^b^	145	112	28	5
virA^b^	142	105	26	11
traG^b^	158	100	39	19
glnA^b^	138	83	34	21
endoD^b^	116	80	26	10
SMU61-homologue^b^	107	78	22	7
sly^b^	99	77	18	4
neuB^bc^	90	77	13	0
srtF-sfp2^b^	90	77	12	1
srtG	76	61	14	1
srtG-sgp2	76	61	14	1
srtG-sgp1	75	60	14	1
adhesinP	43	30	10	3
nadR^c^	25	25	0	0
rgg	33	21	7	5
revS	40	20	9	11
epf ^c^	21	20	1	0
salK^c^	0	0	0	0
salR^c^	0	0	0	0

^a^ Represents VAGs identified in all the isolates

^b^ Represents VAGs tested by chi-square

^c^ Represents lack of VAGs in the commensal pathotype

**Fig. 1:**
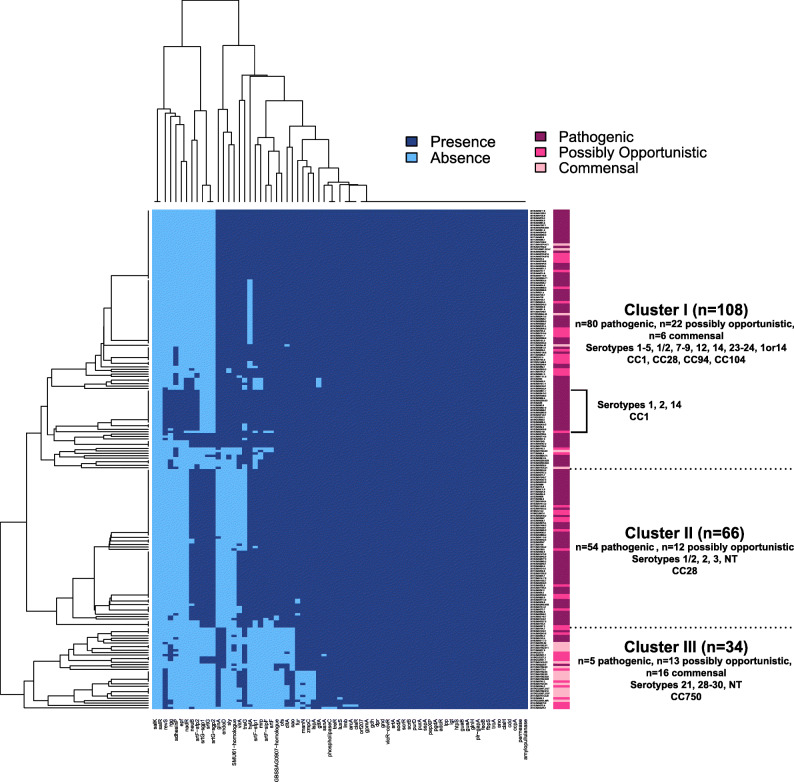
Virulence-associated gene (VAG) profiling of 208 *S. suis* isolates. Heatmap illustrating the presence and absence of 71 previously published VAGs in 208 isolates. Isolates are annotated (right) by pathotype (pathogenic, possibly opportunistic, commensal). Clustering of the 208 *S. suis* isolates by VAGs illustrated three clusters (Clusters I-III), two of which (Clusters II and III) suggest associations between VAGs and pathotype. Serotype and clonal complex (CC) distributions in each of the three clades are also denoted

Clustering analysis was used to determine if relationships between the presence of previously published VAGs and pathotype existed. The analysis of 71 VAGs identified three clusters (Cluster I-III), two of which associated with pathotype (Fig. 1 & Additional file 4). Cluster I consisted of isolates of all three pathotypes. Cluster II predominantly consisted of isolates from the pathogenic pathotype and lacked isolates from the commensal pathotype. Cluster III contained the majority of isolates from the commensal pathotype (73%). Isolates in the pathogenic cluster (Cluster II) were predominantly characterized as serotype 1/2 CC28. Serotype 1/14 CC1 isolates formed a subcluster of Cluster I which lacked isolates from the commensal pathotype. Clustering analysis also illustrated multiple candidate published VAGs for discriminating between pathotypes, specifically VAGs present in the two pathogenic clusters and absent in the commensal cluster.

We then performed statistical analyses to test for associations between VAGs and pathotype. Of the 71 published VAGs detected in the genomes, 16 were tested by chi-square and 14 were considered significant (chi-square *p* < 0.05) (Table 3 & Additional file 5). The classical VAGs *mrp* and *sly* were considered significant by chi-square. The 14 VAGs that were significant by chi-square were further analyzed by the LASSO model. The *sly* gene was in the top ten VAGs identified by LASSO, but *mrp* was not [data not shown]. The LASSO model identified four other candidate VAGs associated with the pathogenic pathotype (Table 4). The VAGs *ofs* and *srtF* were present in over 95% (≥ 132/139) of isolates in the pathogenic pathotype and thus, the presence of both genes was tested as predictors of the pathogenic pathotype. Ninety-five percent (132/139) of the pathogenic pathotype contained both genes while only 23% (5/22) of the commensal pathotype contained both genes.

**Table 4:** LASSO results for the four candidate VAGs in the pathotypes of *S. suis* isolates (*n* = 208)

VAG(s)	No. containing the VAG(s)	Pathogenic (***n*** = 139)		Possibly Opportunistic (***n*** = 47)		Commensal (***n*** = 22)	
		No.	Proportion^**a**^	No.	Proportion^**a**^	No.	Proportion^**a**^
ofs	176	135	0.767	34	0.193	7	0.040
srtF	168	132	0.786	31	0.185	5	0.030
neuB	90	77	0.856	13	0.144	0	0.000
srtF-sfp2	90	77	0.856	12	0.133	1	0.011
ofs and srtF	168	132	0.786	31	0.185	5	0.030

^a^ positive isolates in the pathotype divided by the number of isolates containing the VAG(s)

### Diversity of U.S. *S. suis* by pan-genome analysis

#### Relatedness of 208 *S. suis* isolates by pan-genome analysis

**Fig. 2:**
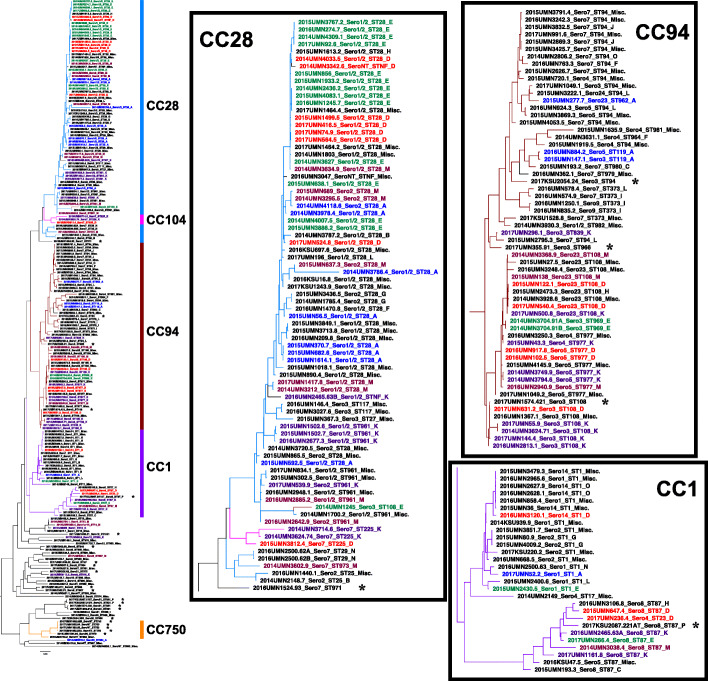
Relatedness of 208 *S. suis* isolates by pan-genome analysis. Genetic relationships between isolates are based on the presence and absence of 8373 gene clusters among 208 *S. suis* genomes. The phylogenetic tree is colored-coded (branches) and labeled (right) by CC; multiple STs did not form a CC or formed a CC without a primary founder. Isolates from at least sixteen swine production companies (A-P) (≥ 2 isolates each) were identified in the data set. Misc. refers to miscellaneous production companies (single isolates each). Isolates belonging to the five predominant production companies (A, D, E, K, and M) are color-coded by their respective production company. * strains in the commensal pathotype (*n* = 22)

Pan-genome analysis of 208 *S. suis* genomes generated a pan-genome of 8373 gene clusters and illustrated multiple clusters that corresponded to the five MLST CCs (CC1, CC28, CC94, CC104, and CC750) (Fig. 2) [28]. Isolates from at least sixteen swine production companies (A-P) (≥ 2 isolates each) were identified in the data set, with A (*n* = 13), D (*n* = 16), E (*n* = 18), K (*n* = 21), and M (*n* = 16) representing the five production companies with the most isolates in this study (predominant production companies). The most predominant CCs (CC1, CC28, and CC94) were identified in multiple production companies. CCs 1, 28, and 94 were identified in 12, 11, and 12 of the 16 production companies, respectively.

#### Relatedness of isolates within the five predominant production companies

**Fig. 3:**
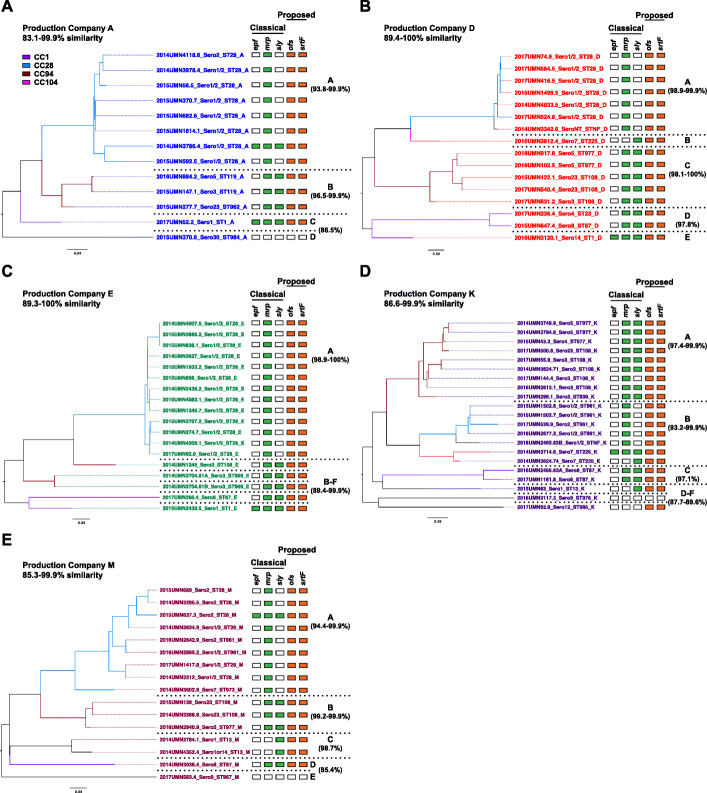
Pan-genome analysis of isolates from the five predominant production companies. The predominant production companies are presented as A, D, E, K, and M. Color-coding of isolate names by production company and color-coding of phylogenetic tree branches by CC follow the same color schemes as Fig. 2. The percent similarity of isolates within a cluster is defined as the percentage of shared genes from a total of 8373 genes. The presence of the classical VAGs *epf*, *mrp*, and *sly* is represented in green and the proposed VAGs *ofs* and *srtF* in orange

The genetic relationships between pathogenic and possibly opportunistic isolates within a production company were investigated using pan-genome analysis for each of the five predominant production companies A, D, E, K, and M (Fig. 3). None of the isolates from these production companies were classified as the commensal pathotype [28]. In addition, we explored associations between pan-genome clusters and genotypes of the classical (*epf*, *mrp*, and *sly*) and proposed pathogenic (*ofs* and *srtF* genes) VAGs. The isolates demonstrated various genotypes of classical VAGs, and a majority (96.4%) possessed the proposed *ofs*+/*srtF*+ genotype for predicting pathogenic strains.

Due to the diversity of isolates within each production company, we investigated pan-genome clusters and genotypes for each predominant production company. The isolates originating from production companies A, E, and M were placed into two, one, and three clusters, respectively, and multiple singletons each and demonstrated an overall 83.1–99.9%, 89.3–100% and 85.3–99.9% similarity, respectively. A majority of the isolates originating from A and E (A = 54%, E = 72%), and many from M (50%) possessed the classical VAG *mrp* but lacked the *epf* and *sly* genes. The isolates originating from production companies D and K were placed into three clusters and multiple singletons each and demonstrated an overall 89.4–100% and 86.6.-99.9% similarity, respectively. Multiple isolates from D and K (D = 44%, K = 48%) possessed *mrp* and *sly* but lacked the *epf* gene. A few isolates from each production company (*n* = 1 from companies D, E, K, and M, *n* = 2 from company A) possessed all three classical VAGs *epf*, *mrp*, and *sly*. A majority of isolates in all five production companies (A = 92.3%, D = 100%, E = 100%, K = 95.2%, M = 93.8%) possessed the proposed *ofs+/srtF+* genotype, indicating the presence of both *ofs* and *srtF* genes are a better predictor of pathogenicity than the presence of the *epf, mrp,* and *sly* genes.

#### Relatedness of commensal isolates

**Fig. 4:**
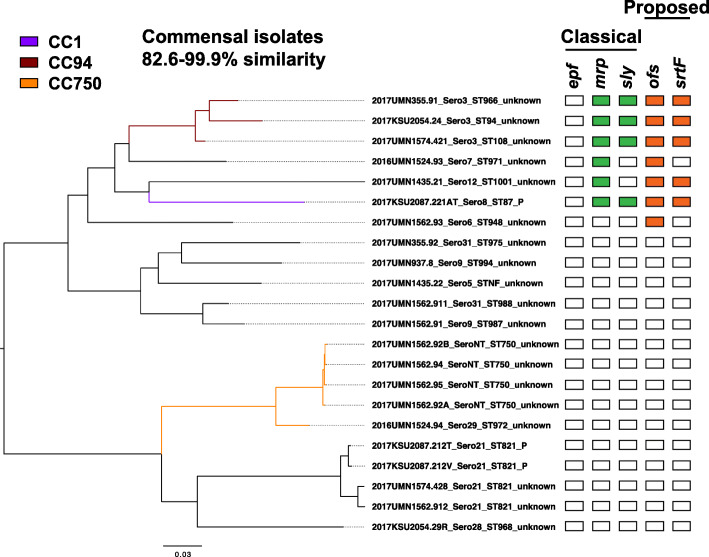
Pan-genome analysis of the 22 commensal isolates. Color-coding of phylogenetic tree branches by CC follows the same color scheme as Fig. 2. The presence of the classical VAGs *epf*, *mrp*, and *sly* is represented in green and the proposed VAGs *ofs* and *srtF* in orange

We further investigated the genetic relationships between the 22 isolates of the commensal pathotype. An 82.6–99.9% similarity was observed, with isolates forming two large clusters and multiple sub-clusters (Fig. 4). Thirteen isolates lacked a CC, while one, three, and five isolates were assigned to CC1, CC94, and CC750, respectively. The CC1 and CC94 isolates possessed more VAGs than the other commensal isolates with all possessing *mrp* and *sly*, and both *ofs* and *srtF,* while a majority of commensal isolates (77.3%) lacked the classical and proposed pathogenic VAGs.

#### Characterization of the validation set of *S. suis* isolates

**Fig. 5:**
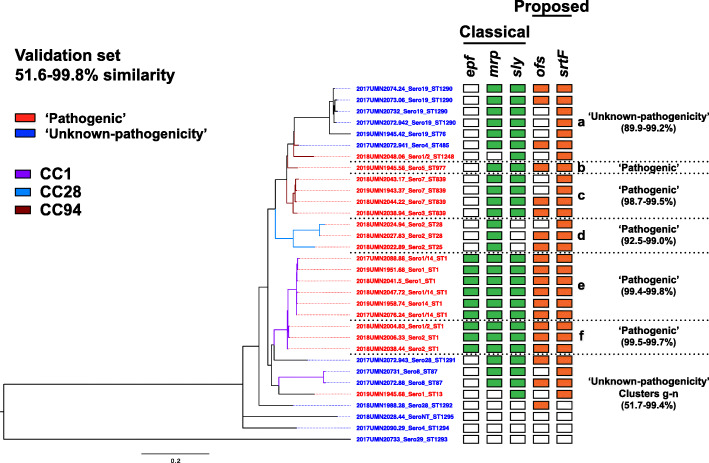
Pan-genome analysis of the validation set of 32 *S. suis* isolates. Isolates are color coded by classification, ‘pathogenic’ (red) or ‘unknown-pathogenicity’ (blue). The phylogenetic tree branches are colored-coded by CC. The percent similarity of isolates within a cluster is defined as the percentage of shared genes from a total of 7078 genes. The presence of the classical VAGs *epf*, *mrp*, and *sly* is represented in green and the proposed VAGs *ofs* and *srtF* in orange

A distinct validation set of 32 *S. suis* isolates was obtained from a single production company to perform pan-genome analysis and further test the novel proposed pathogenic genotype (*ofs+/srtF+*). These isolates were classified as either ‘pathogenic’ or of ‘unknown-pathogenicity.’ The pan-genome consisted of 7078 gene clusters among the 32 genomes, and these pan-genome clusters associated with the ‘pathogenic’ and ‘unknown-pathogenicity’ classifications, as well as with virulence-associated genotypes (Fig. 5). Clusters c-f corresponded to the ‘pathogenic’ classification. Only the isolates in clusters e and f possessed the classical VAGs *epf*, *mrp*, and *sly*. Moreover, all the isolates in these two clusters possessed the proposed pathogenic *ofs+/srtF+* genotype. A majority of the isolates in cluster d (67%) possessed the *ofs+/srtF+* genotype.

Cluster a and singletons g-n corresponded to the ‘unknown-pathogenicity’ classification (Fig. 5). A majority (86%) of the isolates in cluster a possessed the classical VAGs *mrp* and *sly*, but three isolates (43%) also possessed the proposed pathogenic genotype. A majority (75%) of the singletons g-n lacked both the classical and proposed VAGs. Two isolates possessed the proposed pathogenic genotype. The diversity and lack of VAGs in clusters g-n is similar to the diversity seen among the commensal pathotype, suggesting these isolates are commensal strains.

## Discussion

In this study, 71 published *S. suis* VAGs (including the classical VAGs *epf*, *mrp*, *sly*) were evaluated to identify pathogenic isolates associated with systemic and neurological disease from the United States. Notably, VAGs *ofs* and *srtF* demonstrated stronger associations with the pathogenic pathotype than the other 69 VAGs, suggesting novel published VAGs associated with pathogenicity. A genotyping scheme consisting of these two genes (*ofs*+/*srtF*+ genotype) identified pathogenic isolates in a validation set of *S. suis* isolates, demonstrating its potential application for predicting pathogenicity in other swine production companies. The genetic diversity of isolates within and between swine production companies was evaluated by pan-genome analysis, and important associations were observed among pan-genome clusters, CCs, and virulence-associated (VA) genotypes.

Muramidase-released protein has been associated with enhanced survival of *S. suis* in human blood and an increase in blood-brain barrier permeability in mice while suilysin plays a role in the inflammatory response although neither of which have been described as being critical as virulence factors [43–48]. The *epf* gene was identified in only 14% and the *sly* gene was identified in 55% of isolates in the pathogenic pathotype. The *mrp* gene was identified in 91% of the pathogenic pathotype and 27% of the commensal pathotype suggesting the classical VAG *mrp* continues to be an adequate identifier of pathogenic strains. The *epf+*/*mrp*+/*sly*+ genotype is correlated with *S. suis* clinical disease caused by European and Asian ST1 strains belonging to serotypes 1, 2, 9, and 14 [27, 49, 50]. The ST1 isolates in the training set had the *epf+*/*mrp*+/*sly*+ genotype, and the ST25 and ST28 isolates had the *epf*−/*mrp*+/*sly*- genotype, confirming the use of the classical VAGs for identifying virulent ST1 strains but the limited use for identifying ST25 and ST28 strains in North America [11].

Various subtyping methods, including serotyping and MSLT, have been used for evaluating the genetic diversity of *S. suis* isolates and identifying patterns specific to clinical isolates. Pulsed-field gel electrophoresis (PFGE) has been used for evaluating the genetic diversity of *S. suis* serotype 2, 1/2, 3, 7, and 9 strains [51, 52]. Although PFGE has high discriminatory power, typing a large number of isolates is time consuming and labor intensive. Unique randomly amplified polymorphic DNA patterns have been recovered from *S. suis* isolates from diseased pigs and correlated with the production of virulence markers [53, 54]. However, these analyses were mainly focused on serotype 2 strains. Multiplex PCR assays were developed for the differentiation of isolates into serotypes and detection of multiple VAGs [55–57]. A limitation of multiplex PCR assays is the number of targets that can successfully be tested in a single assay [58]. In this study, we utilized pan-genome analysis in conjunction with serotyping, MLST, and VAG profiling as a subtyping tool for *S. suis*. Whole genome sequencing (WGS)-based approaches, such as comparative genome hybridization, minimum core genome sequence typing, pan-genome and Bayesian analysis of population structure, and genome-wide association studies, have been used in combination with phenotypic methods for the identification and classification of *S. suis* strains into groups of differing levels of virulence [21, 22, 59–62]. WGS-based approaches have multiple advantages to molecular subtyping techniques, such as the ability to characterize the entire genome, a higher discriminatory power capable of discriminating closely related strains, the ability to perform in silico (via computer simulation) analyses, and access to a vast number of bioinformatics tools for the analysis of whole genomes [63, 64].

Novel published VAGs for the identification of pathogenic *S. suis* isolates were selected using a chi-square test and a LASSO regression model testing associations between published VAGs and pathotype. As a result, the two genes *ofs* and *srtF* were selected as the ‘best’ indicators of pathogenicity for isolates in our study. The *ofs* gene encodes a serum opacity factor and was associated with virulence attenuation in an experimental pig model [65]. The *srtF* gene encodes a class C sortase and is part of the *srtF* pilus gene cluster composed of four genes, *srtF*, *sipF*, *sfp1*, and *sfp2* [66]*. SrtF* gene mutants of *S. suis* serotype 2 ST1 strain P1/7 caused attenuation of virulence in an intranasal caesarean-derived colostrum-deprived (CDCD) pig model [67]. However, the presence of the pilus gene cluster does not guarantee pilus protein expression [11]. Our research identifies the genes as markers for pathogenicity and not the expression of proteins. The percentage of isolates containing the *ofs*+/*srtF*+ genotype that were classified as pathogenic increased from 79 to 96% (132/137) when excluding the possibly opportunistic pathotype (isolates possibly associated with respiratory disease) from the analysis. The proposed pathogenic genotype for predicting pathogenicity was further tested in a validation set consisting of 32 *S. suis* isolates to evaluate the likelihood of these two genes identifying pathogenic strains in other swine production companies. The *ofs*+/*srtF*+ genotype was observed in 73.7% (14/19) of the ‘pathogenic’ isolates, together indicating a ≥ 74% probability that an isolate will be classified as pathogenic given the proposed genotype. The proposed *ofs*+/*srtF*+ genotype, in complement to the classical VAGs for ST1, identifies pathogenic strains in the United States. A potential application of this research is the development of a diagnostic PCR test targeting these two proposed VAGs.

Nineteen of 139 isolates in the pathogenic pathotype lacked the *ofs*+/*srtF*+ genotype suggesting the possibility of misclassification of these isolates based on tissue source (systemic versus non-systemic). In addition to pathogen-specific traits, environmental and management conditions and host traits contribute to the development of *S. suis* disease. These factors include temperature fluctuations, overcrowding, concurrent infections, and host immunity and genetics [20, 68, 69]. The *ofs*+/*srtF*+ genotype was identified in five isolates in the commensal pathotype but four of these isolates were characterized as CC1 or CC94, which are generally pathogenic subtypes [70, 71]. The five commensal isolates are present in Cluster I (Fig. 1), which represents a cluster containing all three pathotypes, indicating these isolates share similar VAGs with pathogenic isolates. Virulent strains have been previously isolated from the nasal cavities and tonsils of clinically healthy pigs, so characterization by tissue source can be misleading [72, 73].

Pan-genome analysis in combination with metadata (serotype, ST/CC, VA genotype) was used in this study as a subtyping tool to describe the genetic diversity of *S. suis* isolates within a production company and between companies for epidemiological purposes. The differentiation of *S. suis* may provide information on the origin of isolates (geographical location, year, source, etc.) or aid in the identification and tracking of strains over time [74–76]. Isolates from the pathogenic pathotype in this study formed distinct clusters with correlation to CC and VA genotypes, which is consistent with previous studies [54, 77]. The same CC-VA genotype patterns were identified in multiple production companies, suggesting a lack of association between production company, CC, or VA genotype. These observed patterns may be widespread as opposed to originating from a common source of infection as previously suggested [78–80]. Furthermore, the high genetic similarity and identical CC and VAG genotypes within a pan-genome cluster (such as in cluster A in production company E) are indicative of a clone, providing useful information for the identification and tracking of clones over time [81–83]. Thus, the use of WGS to complement metadata (e.g. epidemiological, clinical and demographical data) provides a valuable tool for subtyping *S. suis* as part of epidemiological studies [84, 85]. Further, pan-genome analysis of U.S. *S. suis* isolates may be used to identify candidate VAGs not yet identified or characterized.

The differentiation of *S. suis* isolates is also crucial for the development of autogenous vaccines [86]. Different strains have been recovered from diseased pigs from the same herd and selecting the strain or strains associated with disease is challenging [52, 87–89]. For the validation set, multiple CC-VA genotype patterns were found among the ‘pathogenic’ clusters, indicating multiple clones were present in this production company. This diversity of isolates is supported by the identification of five serotypes (1, 1/2, 2, 14, and 7) in the validation set, all of which are generally pathogenic subtypes [24, 90, 91]. Despite the diversity of clinical strains in the same herd, previous reports indicate a specific strain is the predominant cause of disease and the primary candidate for an autogenous vaccine [87–89, 92]. CC1 was predominantly identified in this production company, and these CC1/ST1 isolates (cluster e and f) demonstrated similar gene content (99% similarity) and genotypes but had different serotypes (serotypes 1/14 vs serotype 2). These results suggest two sub-populations with differences in virulence potential and the need for multiple isolates in a vaccine [93, 94]. On the other hand, the CC28 isolates (cluster d) demonstrated similar gene content (92–99% similarity), serotypes, and genotypes, suggesting similar virulence potential, and the selection of a single isolate for vaccine [11, 13]. As these isolates came from the same production company, all three isolates may by recommended for vaccine development. In addition to the genetic diversity of *S. suis* isolates, historical background of a production company should be considered while selecting isolates. Historical factors such as prior on-farm identification of *S. suis*, historic and current sources of replacement animals, and other confounding disease factors can further support the inclusion of multiple isolates in vaccine development.

## Conclusion

In this study, the current distribution of published, including classical, VAGs in U.S. isolates was determined, which indicated that classical VAGs are not sufficient to differentiate pathogenic and commensal U.S. strains. Of the 71 published VAGs investigated, the *ofs* and *srtF* genes were shown to be stronger predictors of pathogenicity in both a training and a validation set of isolates. Furthermore, a WGS-based approach was used to determine the genetic diversity of isolates demonstrating its use in epidemiological studies and vaccine isolate selection.

## Supplementary Information

**Additional file 1.** Confirmed and putative VAGs of *S. suis* used in this study. Name and accession numbers of the 71 previously published VAGs of *S. suis* used in this study.

**Additional file 2.** Characteristics of the 32 *S. suis* isolates in the validation set. List and identification of the validation set of isolates, including classification, serotype, ST, and SRA accession numbers.

**Additional file 3.** Presence-absence data of the 71 VAGs for the training set of *S. suis* isolates (*n* = 208). Binary matrix representing the presence (1) and absence (0) of the 71 VAGs.

**Additional file 4.** Pathotype, serotype, and ST distribution of clusters I-III identified by VAG profiling. Supporting information for Fig. 1 illustrating the three clusters identified by VAG profiling.

**Additional file 5.** Chi-square and Fisher’s exact test results for the 16 VAGs tested. Chi-square and Fisher’s exact test results for evaluation associations between published *S. suis* VAGs and pathotype.

## Data Availability

The datasets generated and/or analyzed during the current study are available in the Sequence Read Archive. Illumina sequencing data from the training set [28] were deposited under the accession numbers SRR9123061-SRR9123268. Illumina sequencing data from the validation set were deposited under the accession numbers SRR12964194-SRR12964227. The accession numbers from the validation set can also be found in Additional file 2.

## Abbreviations

CC: Clonal complex
*epf*: Extracellular protein factor
LASSO: Least Absolute Shrinkage and Selection Operator
*mrp*: Muramidase-released protein
*ofs*: Serum opacity factor
PFGE: Pulsed-field gel electrophoresis
*sly*: Suilysin
SRST2: Short Read Sequence Typing for Bacterial Pathogens 2
*srtF*: Sortase F
MLST or ST: Multilocus sequence type, sequence type
WGS: Whole genome sequencing
VAG or VA genotype: Virulence-associated gene, virulence-associated genotype

## References

[1] Higgins R, Gottschalk M, Boudreau M, Lebrun A, Henrichsen J (1995). Description of six new capsular types (29–34) of *Streptococcus suis*. J Vet Diagn Investig.

[2] Hill JE, Gottschalk M, Brousseau R, Harel J, Hemmingsen SM, Goh SH (2005). Biochemical analysis, cpn60 and 16S rDNA sequence data indicate that *Streptococcus suis* serotypes 32 and 34, isolated from pigs, are *Streptococcus orisratti*. Vet Microbiol.

[3] Tien LHT, Nishibori T, Nishitani Y, Nomoto R, Osawa R (2013). Reappraisal of the taxonomy of *Streptococcus suis* serotypes 20, 22, 26, and 33 based on DNA–DNA homology and sodA and recN phylogenies. Vet Microbiol.

[4] King SJ, Leigh JA, Heath PJ, Luque I, Tarradas C, Dowson CG, Whatmore AM (2002). Development of a multilocus sequence typing scheme for the pig pathogen *Streptococcus suis*: identification of virulent clones and potential capsular serotype exchange. J Clin Microbiol.

[5] Jolley KA, Bray JE, Maiden MCJ (2018). Open-access bacterial population genomics: BIGSdb software, the PubMLST.org website and their applications. Wellcome Open Res.

[6] Food and Drug Administration Center for Veterinary Medicine (2003). Evaluating the safety of antimicrobial new animal drugs with regard to their microbiological effects on bacteria of human health concern. Guid Ind.

[7] Higgins R, Gottschalk M (2001). Distribution of *Streptococcus suis* capsular types in 2000. Can Vet J Rev Veterinaire Can..

[8] Messier S, Lacouture S, Gottschalk M (2008). Distribution of *Streptococcus suis* capsular types from 2001 to 2007. Can Vet J Rev Veterinaire Can.

[9] Gottschalk M, Lacouture S, Bonifait L, Roy D, Fittipaldi N, Grenier D (2013). Characterization of *Streptococcus suis* isolates recovered between 2008 and 2011 from diseased pigs in Québec, Canada. Vet Microbiol.

[10] Gottschalk M, Lebrun A, Wisselink H, Dubreuil JD, Smith H, Vecht U (1998). Production of virulence-related proteins by Canadian strains of *Streptococcus suis* capsular type 2. Can J Vet Res Rev Can Rech Veterinaire..

[11] Fittipaldi N, Xu J, Lacouture S, Tharavichitkul P, Osaki M, Sekizaki T, Takamatsu D, Gottschalk M (2011). Lineage and virulence of *Streptococcus suis* serotype 2 isolates from North America. Emerg Infect Dis.

[12] Fittipaldi N, Fuller TE, Teel JF, Wilson TL, Wolfram TJ, Lowery DE, Gottschalk M. Serotype distribution and production of muramidase-released protein, extracellular factor and suilysin by field strains of *Streptococcus suis* isolated in the United States. Vet Microbiol. 2009;139:310–7. 10.1016/j.vetmic.2009.06.024 19596529

[13] Athey TBT, Auger J-P, Teatero S, Dumesnil A, Takamatsu D, Wasserscheid J, Dewar K, Gottschalk M, Fittipaldi N. Complex population structure and virulence differences among serotype 2 *S**treptococcus suis* strains belonging to sequence type 28. PLoS One. 2015;10:e0137760. 10.1371/journal.pone.0137760 PMC4574206 26375680

[14] Auger J-P, Fittipaldi N, Benoit-Biancamano M-O, Segura M, Gottschalk M (2016). Virulence studies of different sequence types and geographical origins of *Streptococcus suis* serotype 2 in a mouse model of infection. Pathogens..

[15] Leuwerke B, Wilson K, Schulz K. Control Strategies for Streptococcus suis. Webinar. 2019; https://www.nationalhogfarmer.com/animal-health/strep-suis-control-topic-upcoming-webinar. Accessed 23 Sept 2020.

[16] Pig Health Today. Watch your Strep: keep your guard up for this evolving bacterium. ThePigSite. 2019. https://www.thepigsite.com/news/2019/08/watch-your-strep-keep-your-guard-up-for-this-evolving-bacterium. Accessed 1 Dec 2020.

[17] Baums CG, Brüggemann C, Kock C, Beineke A, Waldmann K-H, Valentin-Weigand P (2010). Immunogenicity of an autogenous *Streptococcus suis* bacterin in preparturient sows and their piglets in relation to protection after weaning. Clin Vaccine Immunol CVI.

[18] Segura M, Fittipaldi N, Calzas C, Gottschalk M (2017). Critical *Streptococcus suis* virulence factors: are they all really critical?. Trends Microbiol.

[19] Galina L, Collins JE, Pijoan C (1992). Porcine *Streptococcus suis* in Minnesota. J Vet Diagn Investig.

[20] Reams RY, Glickman LT, Harrington DD, Thacker HL, Bowersock TL (1994). *Streptococcus suis* infection in swine: a retrospective study of 256 cases. Part II. Clinical signs, gross and microscopic lesions, and coexisting microorganisms. J Vet Diagn Investig.

[21] de Greeff A, Wisselink HJ, de Bree FM, Schultsz C, Baums CG, Thi HN, Stockhofe-Zurwieden N, Smith HE (2011). Genetic diversity of *Streptococcus suis* isolates as determined by comparative genome hybridization. BMC Microbiol.

[22] Zhang A, Yang M, Hu P, Wu J, Chen B, Hua Y, Yu J, Chen H, Xiao J, Jin M (2011). Comparative genomic analysis of *Streptococcus suis* reveals significant genomic diversity among different serotypes. BMC Genomics.

[23] Quessy S, Dubreuil JD, Caya M, Létourneau R, Higgins R (1994). Comparison of pig, rabbit and mouse IgG response to *Streptococcus suis* serotype 2 proteins and active immunization of mice against the infection. Can J Vet Res Rev Can Rech Veterinaire..

[24] Segura M, Aragon V, Brockmeier SL, Gebhart C, de Greeff A, Kerdsin A, O’Dea MA, Okura M, Saléry M, Schultsz C, Valentin-Weigand P, Weinert LA, Wells JM, Gottschalk M. Update on *Streptococcus suis* research and prevention in the era of antimicrobial restriction: 4th International Workshop on *S. suis*. Pathogens. 2020;9:374. 10.3390/pathogens9050374 PMC7281350 32422856

[25] Fittipaldi N, Segura M, Grenier D, Gottschalk M (2012). Virulence factors involved in the pathogenesis of the infection caused by the swine pathogen and zoonotic agent *Streptococcus suis*. Future Microbiol.

[26] Gottschalk M, Segura M (2000). The pathogenesis of the meningitis caused by *Streptococcus suis*: the unresolved questions. Vet Microbiol.

[27] Goyette-Desjardins G, Auger J-P, Xu J, Segura M, Gottschalk M (2014). *Streptococcus suis*, an important pig pathogen and emerging zoonotic agent-an update on the worldwide distribution based on serotyping and sequence typing. Emerg Microbes Infect.

[28] Estrada AA, Gottschalk M, Rossow S, Rendahl A, Gebhart C, Marthaler DG. Serotype and genotype (multilocus sequence type) of *Streptococcus suis* isolates from the United States serve as predictors of pathotype. J Clin Microbiol. 2019;57:e00377–19. 10.1128/JCM.00377-19 PMC6711919 31243086

[29] Inouye M, Dashnow H, Raven L-A, Schultz MB, Pope BJ, Tomita T, Zobel J, Holt KE (2014). SRST2: rapid genomic surveillance for public health and hospital microbiology labs. Genome Med.

[30] R Core Team (2017). R: a language and environment for statistical computing.

[31] Tibshirani R (1996). Regression shrinkage and selection via the Lasso. J R Stat Soc Ser B Methodol.

[32] Friedman J, Hastie T, Tibshirani R (2010). Regularization paths for generalized linear models via coordinate descent. J Stat Softw.

[33] Li D, Luo R, Liu C-M, Leung C-M, Ting H-F, Sadakane K, Yamashita H, Lam T-W (2016). MEGAHIT v1.0: a fast and scalable metagenome assembler driven by advanced methodologies and community practices. Methods San Diego Calif.

[34] Walker BJ, Abeel T, Shea T, Priest M, Abouelliel A, Sakthikumar S, Cuomo CA, Zeng Q, Wortman J, Young SK, Earl AM (2014). Pilon: an integrated tool for comprehensive microbial variant detection and genome assembly improvement. PLoS One.

[35] Gurevich A, Saveliev V, Vyahhi N, Tesler G (2013). QUAST: quality assessment tool for genome assemblies. Bioinformatics..

[36] Seemann T (2014). Prokka: rapid prokaryotic genome annotation. Bioinforma Oxf Engl..

[37] Page AJ, Cummins CA, Hunt M, Wong VK, Reuter S, Holden MTG, Fookes M, Falush D, Keane JA, Parkhill J (2015). Roary: rapid large-scale prokaryote pan genome analysis. Bioinforma Oxf Engl.

[38] van Vliet AHM (2017). Use of pan-genome analysis for the identification of lineage-specific genes of *Helicobacter pylori*. FEMS Microbiol Lett.

[39] Athey TBT, Teatero S, Lacouture S, Takamatsu D, Gottschalk M, Fittipaldi N (2016). Determining *Streptococcus suis* serotype from short-read whole-genome sequencing data. BMC Microbiol.

[40] Meng P, Lu C, Zhang Q, Lin J, Chen F (2017). Exploring the genomic diversity and cariogenic differences of *Streptococcus mutans* strains through pan-genome and comparative genome analysis. Curr Microbiol.

[41] Medini D, Donati C, Tettelin H, Masignani V, Rappuoli R (2005). The microbial pan-genome. Curr Opin Genet Dev.

[42] Li M, Wang C, Feng Y, Pan X, Cheng G, Wang J, Ge J, Zheng F, Cao M, Dong Y, Liu D, Wang J, Lin Y, Du H, Gao GF, Wang X, Hu F, Tang J (2008). SalK/SalR, a two-component signal transduction system, is essential for full virulence of highly invasive *Streptococcus suis* serotype 2. PLoS One.

[43] Pian Y, Li X, Zheng Y, Wu X, Yuan Y, Jiang Y (2016). Binding of human fibrinogen to MRP enhances *Streptococcus suis* survival in host blood in a αXβ2 integrin-dependent manner. Sci Rep.

[44] Wang J, Kong D, Zhang S, Jiang H, Zheng Y, Zang Y, Hao H, Jiang Y (2015). Interaction of fibrinogen and muramidase-released protein promotes the development of *Streptococcus suis* meningitis. Front Microbiol.

[45] Tenenbaum T, Asmat TM, Seitz M, Schroten H, Schwerk C (2016). Biological activities of suilysin: role in *Streptococcus suis* pathogenesis. Future Microbiol.

[46] Allen AG, Bolitho S, Lindsay H, Khan S, Bryant C, Norton P, Ward P, Leigh J, Morgan J, Riches H, Eastty S, Maskell D (2001). Generation and characterization of a defined mutant of *Streptococcus suis* lacking suilysin. Infect Immun.

[47] Lun S, Perez-Casal J, Connor W, Willson PJ (2003). Role of suilysin in pathogenesis of *Streptococcus suis* capsular serotype 2. Microb Pathog.

[48] Smith HE, Vecht U, Wisselink HJ, Stockhofe-Zurwieden N, Biermann Y, Smits MA. Mutants of *Streptococcus suis* types 1 and 2 impaired in expression of muramidase-released protein and extracellular protein induce disease in newborn germfree pigs. Infect Immun. 1996;64:4409–12. 10.1128/iai.64.10.4409-4412.1996 PMC174391 8926123

[49] Wisselink HJ, Smith HE, Stockhofe-Zurwieden N, Peperkamp K, Vecht U (2000). Distribution of capsular types and production of muramidase-released protein (MRP) and extracellular factor (EF) of *Streptococcus suis* strains isolated from diseased pigs in seven European countries. Vet Microbiol.

[50] Wei Z, Li R, Zhang A, He H, Hua Y, Xia J, Cai X, Chen H, Jin M (2009). Characterization of *Streptococcus suis* isolates from the diseased pigs in China between 2003 and 2007. Vet Microbiol.

[51] Berthelot-Hérault F, Marois C, Gottschalk M, Kobisch M (2002). Genetic diversity of *Streptococcus suis* strains isolated from pigs and humans as revealed by pulsed-field gel electrophoresis. J Clin Microbiol.

[52] Vela AI, Goyache J, Tarradas C, Luque I, Mateos A, Moreno MA, Borge C, Perea JA, Dominguez L, Fernandez-Garayzabal JF (2003). Analysis of genetic diversity of *Streptococcus suis* clinical isolates from pigs in Spain by pulsed-field gel electrophoresis. J Clin Microbiol.

[53] Chatellier S, Gottschalk M, Higgins R, Brousseau R, Harel J (1999). Relatedness of *Streptococcus suis* serotype 2 isolates from different geographic origins as evaluated by molecular fingerprinting and phenotyping. J Clin Microbiol.

[54] Maneerat K, Yongkiettrakul S, Kramomtong I, Tongtawe P, Tapchaisri P, Luangsuk P, Chaicumpa W, Gottschalk M, Srimanote P (2013). Virulence genes and genetic diversity of *Streptococcus suis* serotype 2 isolates from Thailand. Transbound Emerg Dis.

[55] Silva LMG, Baums CG, Rehm T, Wisselink HJ, Goethe R, Valentin-Weigand P (2006). Virulence-associated gene profiling of *Streptococcus suis* isolates by PCR. Vet Microbiol.

[56] Kerdsin A, Akeda Y, Hatrongjit R, Detchawna U, Sekizaki T, Hamada S, Gottschalk M, Oishi K (2014). *Streptococcus suis* serotyping by a new multiplex PCR. J Med Microbiol.

[57] Okura M, Lachance C, Osaki M, Sekizaki T, Maruyama F, Nozawa T, Nakagawa I, Hamada S, Rossignol C, Gottschalk M, Takamatsu D (2014). Development of a two-step multiplex PCR assay for typing of capsular polysaccharide synthesis gene clusters of *Streptococcus suis*. J Clin Microbiol.

[58] Yang S, Rothman RE (2004). PCR-based diagnostics for infectious diseases: uses, limitations, and future applications in acute-care settings. Lancet Infect Dis.

[59] Chen C, Zhang W, Zheng H, Lan R, Wang H, Du P, Bai X, Ji S, Meng Q, Jin D, Liu K, Jing H, Ye C, Gao GF, Wang L, Gottschalk M, Xu J (2013). Minimum core genome sequence typing of bacterial pathogens: a unified approach for clinical and public health microbiology. J Clin Microbiol.

[60] Weinert LA, Chaudhuri RR, Wang J, Peters SE, Corander J, Jombart T, Baig A, Howell KJ, Vehkala M, Välimäki N, Harris D, Chieu TTB, Van Vinh CN, Campbell J, Schultsz C, Parkhill J, Bentley SD, Langford PR, Rycroft AN, Wren BW, Farrar J, Baker S, Hoa NT, Holden MTG, Tucker AW, Maskell DJ (2015). Genomic signatures of human and animal disease in the zoonotic pathogen *Streptococcus suis*. Nat Commun.

[61] Willemse N, Howell KJ, Weinert LA, Heuvelink A, Pannekoek Y, Wagenaar JA, Smith HE, van der Ende A, Schultsz C (2016). An emerging zoonotic clone in the Netherlands provides clues to virulence and zoonotic potential of *Streptococcus suis*. Sci Rep.

[62] Wileman TM, Weinert LA, Howell KJ, Wang J, Peters SE, Williamson SM, Wells JM, Langford PR, Rycroft AN, Wren BW, Maskell DJ, Tucker AW. Pathotyping the zoonotic pathogen *Streptococcus suis*: novel genetic markers to differentiate invasive disease-associated isolates from non-disease-associated isolates from England and Wales. J Clin Microbiol. 2019;57:e01712-18. 10.1128/JCM.01712-18 PMC6595460 30944194

[63] Quainoo S, Coolen JPM, van Hijum SAFT, Huynen MA, Melchers WJG, van Schaik W, Wertheim HFL (2017). Whole-genome sequencing of bacterial pathogens: the future of nosocomial outbreak analysis. Clin Microbiol Rev.

[64] Uelze L, Grützke J, Borowiak M, Hammerl JA, Juraschek K, Deneke C, Tausch SH, Malorny B (2020). Typing methods based on whole genome sequencing data. One Health Outlook.

[65] Baums CG, Kaim U, Fulde M, Ramachandran G, Goethe R, Valentin-Weigand P (2006). Identification of a novel virulence determinant with serum opacification activity in *Streptococcus suis*. Infect Immun.

[66] Fittipaldi N, Takamatsu D, de la Cruz Domínguez-Punaro M, Lecours M-P, Montpetit D, Osaki M, Sekizaki T, Gottschalk M (2010). Mutations in the gene encoding the ancillary pilin subunit of the *Streptococcus suis* srtF cluster result in pili formed by the major subunit only. PLoS One.

[67] Faulds-Pain A, Shaw HA, Terra VS, Kellner S, Brockmeier SL, Wren BW (2019). The *Streptococcos suis* sortases SrtB and SrtF are essential for disease in pigs. Microbiol Read Engl.

[68] Dee SA, Carlson AR, Winkelman NL, Corey MM (1993). Effect of management practices on the *Streptococcus suis* carrier rate in nursery swine. J Am Vet Med Assoc.

[69] Segura M, Calzas C, Grenier D, Gottschalk M (2016). Initial steps of the pathogenesis of the infection caused by *Streptococcus suis*: fighting against nonspecific defenses. FEBS Lett.

[70] Hatrongjit R, Fittipaldi N, Gottschalk M, Kerdsin A. Tools for molecular epidemiology of *Streptococcus suis*. Pathogens. 2020;9:81. 10.3390/pathogens9020081 PMC7168656 32012668

[71] Kerdsin A, Akeda Y, Takeuchi D, Dejsirilert S, Gottschalk M, Oishi K. Genotypic diversity of *Streptococcus suis* strains isolated from humans in Thailand. Eur J Clin Microbiol Infect Dis Off Publ Eur Soc Clin Microbiol. 2018;37:917–25. 10.1007/s10096-018-3208-8 29417311

[72] Brisebois LM, Charlebois R, Higgins R, Nadeau M (1990). Prevalence of *Streptococcus suis* in four to eight week old clinically healthy piglets. Can J Vet Res Rev Can Rech Veterinaire.

[73] Marois C, Le Devendec L, Gottschalk M, Kobisch M (2007). Detection and molecular typing of *Streptococcus suis* in tonsils from live pigs in France. Can J Vet Res Rev Can Rech Veterinaire..

[74] Beaudoin M, Harel J, Higgins R, Gottschalk M, Frenette M, MacInnes JI (1992). Molecular analysis of isolates of *Streptococcus suis* capsular type 2 by restriction-endonuclease-digested DNA separated on SDS-PAGE and by hybridization with an rDNA probe. J Gen Microbiol.

[75] Doto DS, Moreno LZ, Calderaro FF, Matajira CEC, de Moura Gomes VT, Ferreira TSP, Mesquita RE, Timenetsky J, Gottschalk M, Moreno AM (2016). Genetic diversity of *Streptococcus suis* serotype 2 isolated from pigs in Brazil. Can J Vet Res Rev Can Rech Veterinaire..

[76] Dutkiewicz J, Sroka J, Zając V, Wasiński B, Cisak E, Sawczyn A, Kloc A, Wójcik-Fatla A (2017). *Streptococcus suis*: a re-emerging pathogen associated with occupational exposure to pigs or pork products. Part I - epidemiology. Ann Agric Environ Med AAEM.

[77] Dong W, Zhu Y, Ma Y, Ma J, Zhang Y, Yuan L, Pan Z, Wu Z, Yao H. Multilocus sequence typing and virulence genotyping of *Streptococcus suis* serotype 9 isolates revealed high genetic and virulence diversity. FEMS Microbiol Lett. 2017;364. 10.1093/femsle/fnx192. 10.1093/femsle/fnx192 29029051

[78] Mwaniki CG, Robertson ID, Trott DJ, Atyeo RF, Lee BJ, Hampson DJ (1994). Clonal analysis and virulence of Australian isolates of *Streptococcus suis* type 2. Epidemiol Infect.

[79] Morales B, Ruiz Á, Lacouture S, Gottschalk M (2015). Clonal distribution of *Streptococcus suis* isolated from diseased pigs in the central region of Chile. Can J Vet Res Rev Can Rech Veterinaire..

[80] Denich LC, Farzan A, Friendship R, Arndt E, Gottschalk M, Poljak Z. A case-control study to investigate the serotypes of *S. suis* isolates by multiplex PCR in nursery pigs in Ontario, Canada. Pathogens. 2020;9:44. 10.3390/pathogens9010044 PMC7168631 31948073

[81] van Belkum A, Tassios PT, Dijkshoorn L, Haeggman S, Cookson B, Fry NK, Fussing V, Green J, Feil E, Gerner-Smidt P, Brisse S, Struelens M, European Society of Clinical Microbiology and Infectious Diseases (ESCMID) Study Group on Epidemiological Markers (ESGEM) (2007). Guidelines for the validation and application of typing methods for use in bacterial epidemiology. Clin Microbiol Infect Off Publ Eur Soc Clin Microbiol Infect Dis.

[82] Allgaier A, Goethe R, Wisselink HJ, Smith HE, Valentin-Weigand P (2001). Relatedness of *Streptococcus suis* isolates of various serotypes and clinical backgrounds as evaluated by macrorestriction analysis and expression of potential virulence traits. J Clin Microbiol.

[83] Du P, Zheng H, Zhou J, Lan R, Ye C, Jing H, Jin D, Cui Z, Bai X, Liang J, Liu J, Xu L, Zhang W, Chen C, Xu J (2017). Detection of multiple parallel transmission outbreak of *Streptococcus suis* human infection by use of genome epidemiology, China, 2005. Emerg Infect Dis.

[84] Rantsiou K, Kathariou S, Winkler A, Skandamis P, Saint-Cyr MJ, Rouzeau-Szynalski K, Amézquita A (2018). Next generation microbiological risk assessment: opportunities of whole genome sequencing (WGS) for foodborne pathogen surveillance, source tracking and risk assessment. Int J Food Microbiol.

[85] Van Goethem N, Descamps T, Devleesschauwer B, Roosens NHC, Boon NAM, Van Oyen H, Robert A (2019). Status and potential of bacterial genomics for public health practice: a scoping review. Implement Sci IS.

[86] Rieckmann K, Pendzialek S-M, Vahlenkamp T, Baums CG (2020). A critical review speculating on the protective efficacies of autogenous *Streptococcus suis* bacterins as used in Europe. Porc Health Manag.

[87] Mogollon JD, Pijoan C, Murtaugh MP, Collins JE, Cleary PP (1991). Identification of epidemic strains of *Streptococcus suis* by genomic fingerprinting. J Clin Microbiol.

[88] Reams RY, Harrington DD, Glickman LT, Thacker HL, Bowersock TL (1996). Multiple serotypes and strains of *Streptococcus suis* in naturally infected swine herds. J Vet Diagn Investig Off Publ Am Assoc Vet Lab Diagn Inc.

[89] Martinez G, Harel J, Lacouture S, Gottschalk M (2002). Genetic diversity of *Streptococcus suis* serotypes 2 and 1/2 isolates recovered from carrier pigs in closed herds. Can J Vet Res Rev Can Rech Veterinaire..

[90] Boetner AG, Binder M, Bille-Hansen V (1987). *Streptococcus suis* infections in Danish pigs and experimental infection with Streptococcus suis serotype 7. Acta Pathol Microbiol Scand Ser B Microbiol.

[91] Smith HE, van Bruijnsvoort L, Buijs H, Wisselink HJ, Smits MA (1999). Rapid PCR test for *Streptococcus suis* serotype 7. FEMS Microbiol Lett.

[92] Torremorell M, Pijoan C (1998). Prolonged persistence of an epidemic *Streptococcus suis* strain in a closed pig population. Vet Rec.

[93] Haesebrouck F, Pasmans F, Chiers K, Maes D, Ducatelle R, Decostere A (2004). Efficacy of vaccines against bacterial diseases in swine: what can we expect?. Vet Microbiol.

[94] Hopkins D, Poljak Z, Farzan A, Friendship R. Field studies evaluating the direct, indirect, total, and overall efficacy of *Streptococcus suis *autogenous vaccine in nursery pigs. Can Vet J Rev Veterinaire Can. 2019;60:386–90. PMC6417621 30992594

